# The prevalence of sarcopenia and risk factors in the older adult in China: a systematic review and meta-analysis

**DOI:** 10.3389/fpubh.2024.1415398

**Published:** 2024-08-05

**Authors:** Shilong Meng, Xiaomin He, Xinlei Fu, Xu Zhang, Minghao Tong, Wei Li, Wei Zhang, Xiaolin Shi, Kang Liu

**Affiliations:** ^1^The Second School of Clinical Medicine, Zhejiang Chinese Medical University, Hangzhou, Zhejiang, China; ^2^The Second Affiliated Hospital, Zhejiang Chinese Medical University, Hangzhou, Zhejiang, China; ^3^The First School of Clinical Medicine, Fujian University of Traditional Chinese Medical, Fuzhou, Fujian, China; ^4^Xianju Branch of the Second Affiliated Hospital, Zhejiang Chinese Medicine University, Taizhou, Zhejiang, China

**Keywords:** sarcopenia, prevalence, risk factors, older adult, Chinese, meta-analysis

## Abstract

**Background:**

Understanding the epidemiological information of a certain disease is the first step in related prevention and control work. This article aims to understand the prevalence and associated risk factors of sarcopenia among the older adult (≥60 years old) in China and to provide an evidence-based basis for early identification, management, and prevention of sarcopenia patients.

**Methods:**

We searched seven databases: CNKI, Wanfang, VIP, PubMed, Web of Science, Embase, and Cochrane Library databases from the establishment of the database until January 31, 2024. The Quality evaluation criteria of cross-sectional studies recommended by the Agency for Healthcare Research and Quality (AHRQ) were used for literature quality evaluation. Stata 18.0 software was used for statistical analysis.

**Results:**

We finally included 45 studies, involving a total of 37,571 cases. After statistical analysis, we found that the prevalence of sarcopenia among the older adult in China was 20.7% [95% CI (18.3, 23.0%)]. The results of subgroup analysis suggest that: ① According to gender, the prevalence rate of sarcopenia in women (21.6%) is higher than that in men (19.2%); ② According to age, the prevalence rate of older adult people aged ≥80 (45.4%) was the highest, followed by 70–79 (27.2%) and 60–69 (15.7%). ③ According to region, the prevalence rate of the older adult in the south (21.7%) is higher than that in the north (19.0%); ④ According to the time of publication, the prevalence of sarcopenia among the older adult in China has increased (from 19.2% in 2014–2018 to 21.4% in 2019–2024); ⑤ According to the diagnostic criteria, the detection rate of AWGS (2019) is higher than that of AWGS (2014) (24.5% vs. 19.3%). Finally, aging, low BMI, low leg circumference, smoking, depression, osteoporosis, malnutrition and malnutrition risk are all risk factors for sarcopenia among the older adult in China.

**Conclusion:**

The prevalence of sarcopenia in the older adult in China was higher (20.7%), which should be paid attention to by relevant health authorities. In addition, aging, low BMI, low calf circumference, smoking, depression, osteoporosis, malnutrition and malnutrition risk are risk factors for the development of sarcopenia in the older adult in China. For these high-risk populations, early identification, prevention, and intervention can be carried out to delay the occurrence and progression of sarcopenia.

## 1 Introduction

Sarcopenia is a common senile disease, which refers to the symptoms of a decline in skeletal muscle mass and muscle strength caused by aging (1). Sarcopenia is highly associated with a variety of adverse outcomes (such as fractures, cognitive decline, metabolic disorders, etc.), and its development process is generally hidden and not easily detected by patients until the occurrence of the above-mentioned adverse consequences (2, 3). Sarcopenia not only seriously affects the quality of life of the older adult, but the follow-up medical care will also bring a heavy economic burden to the family and society (4). An epidemiological study in South Korea showed that the prevalence of sarcopenia in the older adult aged 60 years and above was about 13.1% (5); Japan also reported that the prevalence of sarcopenia in the older adult aged 60 years and above was 9.9% (6). A global epidemiological study of sarcopenia shows that: sarcopenia seriously affects the quality of life of 10–16% of the older adult worldwide (7).

Sarcopenia is an aging disease, and with the global trend of the aging population, the phenomenon of sarcopenia in the older adult will become increasingly common (8, 9). As is well known, China has entered a stage of rapid population aging. According to the results of the 7th National Population Census (10): the population aged 60 and above in China is approximately 264 million, accounting for 18.7% of the total population. At present, sarcopenia has received attention from various countries, but a unified diagnostic standard has not yet been established (8, 11). Among them, the most commonly used diagnostic criteria include the European Working Group on Sarcopenia in Older People (EWGSOP), the Asian Working Group on Sarcopenia (AWGS), and the International Working Group on Sarcopenia (IWGS) (12–15). These diagnostic criteria all recognize that muscle mass, muscle strength, and daily activity ability are the three important factors for diagnosing sarcopenia. However, due to significant differences in factors such as country, region, diet, environment, and race, accurately assessing the epidemiological situation of sarcopenia and carrying out related prevention and treatment work still pose certain challenges (16).

Collecting epidemiological evidence of sarcopenia in the older adult is the first step to formulating preventive procedures or health care services. In the case of insufficient literature reports, systematic evaluation of prevalence and risk factors data is becoming more and more important for policy formulation and implementation of preventive measures. At present, we find that China has begun to pay attention to the epidemiological information of sarcopenia in the older adult, but the conclusions on its epidemiological characteristics, risk factors, and complications are not satisfactory (17–19). At the same time, in the past few years, several large-scale sample studies have been published at home and abroad, which can be used for systematic review and meta-analysis. Therefore, we plan to assess the prevalence and risk factors of sarcopenia among the older adult in China aged 60 and above in China, and provide evidence-based evidence for early identification, management and prevention of sarcopenia among the older adult in China.

## 2 Methods

### 2.1 Search strategy

This study is based on the PRISMA statement (20). Because this study is an epidemiological survey, ethical approval is not required. The systematic review and meta-analysis have been registered in PROSPERO with the number CRD42023494338. We used the following principles of the PICOS algorithm to guide the initial retrieval:

P (population): China older adult people over 60 years old;
I (intervention): no intervention;
C (comparison): no comparison;
O (outcome): the prevalence and risk factors of sarcopenia among the older adult in China;
S (study): cross-sectional study/retrospective study.

### 2.2 Literature search

We searched seven databases: CNKI, Wanfang, VIP, PubMed, Web of Science, Embase, and Cochrane Library from the establishment of the database until January 31, 2024. The method of “subject word + the free word” was used to search, and the Chinese search terms were “sarcopenia”; “prevalence, incidence, epidemiology”; “Influencing factors, related factors, risk factors”; English search terms are: “sarcopenia”; “prevalence, incidence, epidemiology”; “risk factor, correlation factor, affecting factor”; “China, Chinese.”

**Figure 1:**
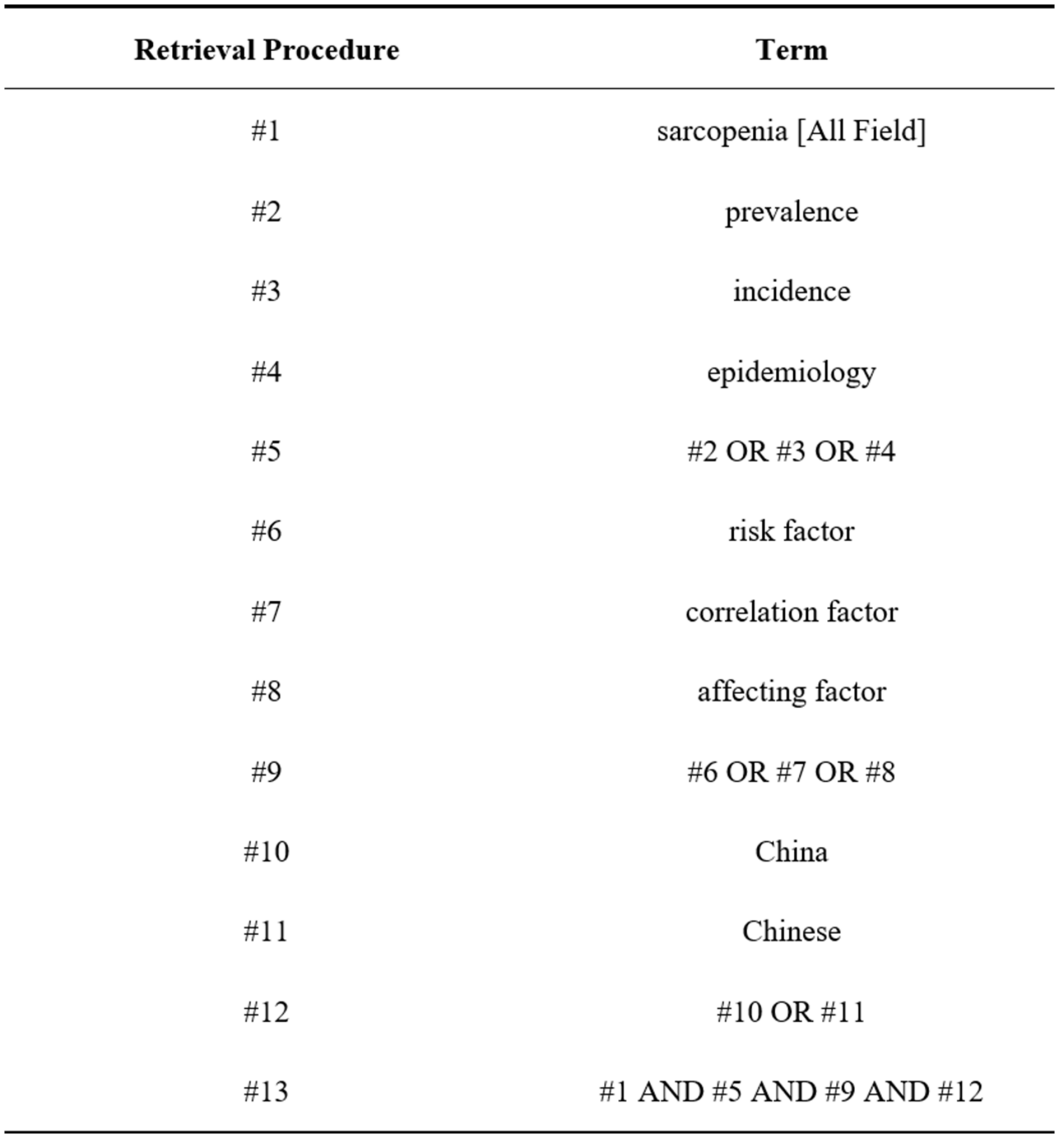
PubMed retrieval procedure flowchart.

The literature retrieval strategy takes PubMed as an example. The detailed retrieval formula of PubMed is as follows: (“sarcopenia”[MeSH Terms] OR “sarcopenia”[All Fields] OR “sarcopenia s”[All Fields]) AND (“epidemiology”[MeSH Subheading] OR “epidemiology”[All Fields] OR “prevalence”[All Fields] OR “prevalence”[MeSH Terms] OR “prevalance”[All Fields] OR “prevalences”[All Fields] OR “prevalence s”[All Fields] OR “prevalent”[All Fields] OR “prevalently”[All Fields] OR “prevalents”[All Fields] OR (“epidemiology”[MeSH Subheading] OR “epidemiology”[All Fields] OR “incidence”[All Fields] OR “incidence”[MeSH Terms] OR “incidences”[All Fields] OR “incident”[All Fields] OR “incidents”[All Fields]) OR (“epidemiologies”[All Fields] OR “epidemiology”[MeSH Subheading] OR “epidemiology”[All Fields] OR “epidemiology”[MeSH Terms] OR “epidemiology s”[All Fields])) AND ((“risk factors”[MeSH Terms] OR (“risk”[All Fields] AND “factors”[All Fields]) OR “risk factors”[All Fields]) AND ((“correlate”[All Fields] OR “correlated”[All Fields] OR “correlates”[All Fields] OR “correlating”[All Fields] OR “correlation”[All Fields] OR “correlation s”[All Fields] OR “correlations”[All Fields] OR “correlative”[All Fields] OR “correlatives”[All Fields]) AND (“factor”[All Fields] OR “factor s”[All Fields] OR “factors”[All Fields])) AND ((“affect”[MeSH Terms] OR “affect”[All Fields] OR “affects”[All Fields] OR “affected”[All Fields] OR “affecteds”[All Fields] OR “affecting”[All Fields]) AND (“factor”[All Fields] OR “factor s”[All Fields] OR “factors”[All Fields]))) AND (“china”[MeSH Terms] OR “china”[All Fields] OR “china s”[All Fields] OR “chinas”[All Fields] OR (“chineses”[All Fields] OR “east asian people”[MeSH Terms] OR (“east”[All Fields] AND “asian”[All Fields] AND “people”[All Fields]) OR “east asian people”[All Fields] OR “chinese”[All Fields])). (For the specific search process, see Figure 1 and Supplementary File 1).

### 2.3 Literature inclusion and exclusion criteria

#### 2.3.1 Inclusion criteria

① According to the author’s language ability, literature in English, and Chinese were eligible; ② The study subjects include the older adult population aged ≥60 in China; ③ The study includes risk factors that may lead to sarcopenia in the older adult; ④ The definition and diagnostic criteria for sarcopenia were proposed in the study; ⑤ The study provided data on the prevalence of sarcopenia, or data that can be used to calculate the prevalence; ⑥ OR values and 95% CI were provided in the study; and ⑦ Cross-sectional or retrospective studies.

#### 2.3.2 Exclusion criteria

① Repeated published research; ② Research published in the form of reviews, conference abstracts, etc.; ③ Research that cannot obtain full text or extract complete data; ④ The sample size of the older adult is too small (≤100 studies); and ⑤ Low-quality research (AHRQ score < 6 points).

### 2.4 Literature screening and data extraction

We import the retrieved literature into EndNoteX9.1 software and first remove the duplicate literature. The second step is to make a preliminary screening by reading the titles and abstracts of the literature. Finally, read the rest of the literature in full, and make the final inclusion and exclusion in strict accordance with the inclusion and exclusion criteria. In this process, two evaluators (W.L. and X.Z.) independently cross-checked the included literature repeatedly. If there is any dispute, it will be resolved through discussion between the two parties or the introduction of the third researcher (XL.F.) for review.

In the data extraction stage, two researchers (W.L. and X.Z.) independently used Excel tables to extract data. The main contents of the extraction are as follows: (1) Basic information included in the literature: first author, publication year, investigation place, etc. (2) Calculate the prevalence rate of sarcopenia and related data of risk factors; (3) Key information of biased risk assessment. After the data is extracted, it shall be summarized, exchanged, and reviewed. If there is disagreement, it shall be submitted to the third researcher (XL.F.) for review.

### 2.5 Literature quality evaluation

We adopt the cross-sectional study quality evaluation criteria recommended by the Agency for Healthcare Research and Quality (AHRQ) for literature quality evaluation (21). The evaluation criteria consist of 11 items, with a rating scale of 1 point for “yes” and 0 points for “no/unclear.” A total score of 8–11 indicates high quality, 4–7 indicates moderate quality, and 0–3 indicates low quality (see Table 1 for details).

**Table 1:** AHRQ cross-sectional study quality evaluation standard table.

	Item content	Yes	No/Unclear
1	Define the source of information (survey or record review?)		
2	List inclusion and exclusion criteria for exposed and unexposed subjects (cases and control) or refer to previous publications		
3	Indicate period used for identifying patients		
4	Indicate whether or not subjects were consecutive if not population-based		
5	Indicate if evaluators of subjective components of the study were masked to other aspects of the status of the participants		
6	Describe any assessments undertaken for quality assurance purposes (e.g., test/retest of primary outcome measurements)		
7	Explain any patient exclusions from the analysis		
8	Describe how confounding was assessed and/or controlled		
9	lf applicable, explain how missing data were handled in the analysis		
10	Summarize patient response rates and completeness of data collection		
11	Clarify what follow-up, if any, was expected and the percentage of patients for which incomplete data or follow-up was obtained		

### 2.6 Statistics

We used Stata18.0 software to statistically analyze the data on the prevalence rate and risk factors of sarcopenia and analyzed the heterogeneity of the included studies through the *Q* test and *I*^2^ value. If *I^2^* > 50%, and *p* < 0.1, it shows that there is high heterogeneity among the studies, and the random effect model is used for analysis. Otherwise, the fixed effect model will be used for statistical analysis (22). If the heterogeneity between the included research results is large, it is necessary to further analyze the sources of heterogeneity, and the subgroup analysis method can be used to try to find out the obvious sources of heterogeneity. The subgroups set in this paper include: gender, age, region, publication time, and diagnostic criteria, and the statistical significance *p*-value in all statistical analyses is set to 0.05.

In this study, we did not check the publication bias. The reasons are as follows: publication bias is a phenomenon in which studies with significant results are easier to publish than those with insignificant results, which may lead to systematic differences between published and unpublished studies (23). However, in the observational study of prevalence, there are no significant or insignificant results, and it is not recommended to use mature methods to test this deviation in the systematic evaluation of prevalence research. Therefore, we did not check the publication bias.

## 3 Results

### 3.1 Literature screening process and results

**Figure 2:**
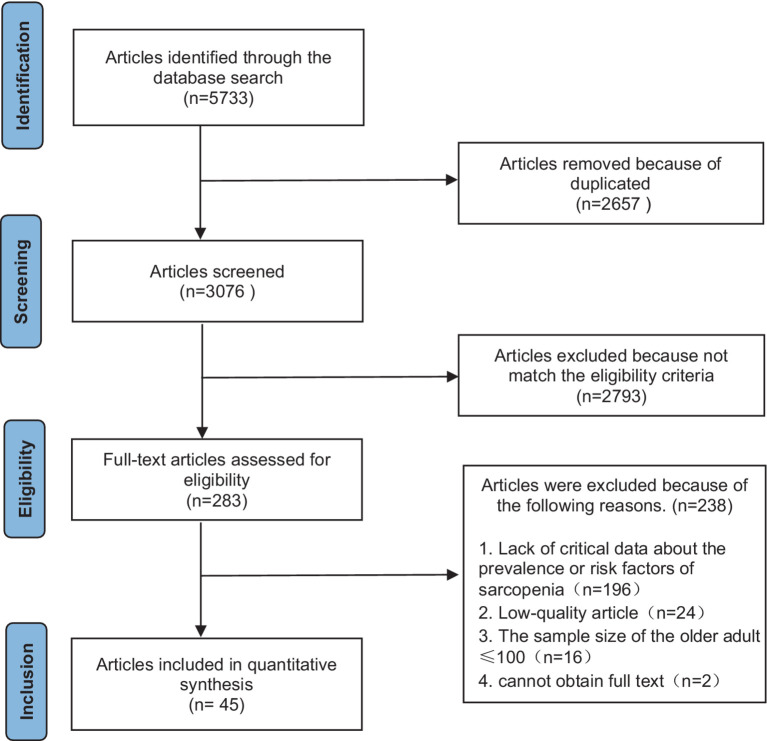
Literature retrieval process diagram.

After a preliminary search, we obtained a total of 5,733 related articles, and after a layer-by-layer screening, we finally included 45 studies, with a total of 37,571 subjects. (For the process and results of literature screening, see Figure 2).

### 3.2 Basic characteristics and quality evaluation results of included literature

To ensure the quality of the included literature, we set the inclusion quality standard (AHRQ Score ≥ 6). A total of 45 literatures were included in this study, involving 37,571 older adult people over 60 years old in China in 31 provinces and cities. The general situation and quality evaluation results of the included literature are shown in Table 2. (For the detailed results of literature evaluation, see Supplementary File 2; for the general information of literature, see Supplementary File 3).

**Table 2:** General information and quality evaluation results of the included literature.

	Study	Region	Population source	Diagnostic criteria	Muscle mass assessment methods	Sarcopenia size	Sample size	AHRQ score
1	Wang et al. 2024 (24)	Yunnan-guizhou Plateau region*	Community population	AWGS (2019)	BIA	194	1,327	8
2	Dai et al. 2023 (25)	China	Community population	AWGS (2019)	BIA	919	5,016	6
3	Wan et al. 2023 (26)	Gansu	Inpatients	AWGS (2019)	BIA	232	540	6
4	Zou et al. 2023 (27)	Anhui	Community population	AWGS (2019)	BIA	214	1716	6
5	Ma et al. 2023 (28)	Shandong	Inpatients	AWGS (2019)	BIA	143	606	6
6	Chen et al. 2023 (29)	Xiangtan	Community population	AWGS (2019)	BIA	87	556	7
7	Musha et al. 2023 (30)	Xinjiang	Community population	AWGS (2019)	BIA	184	1,561	7
8	He et al. 2022 (31)	Shanghai	Community population	AWGS (2019)	BIA	275	1,407	9
9	Zhong et al. 2022 (32)	Hunan	Community population	AWGS (2019)	BIA	282	1,040	7
10	Tang et al. 2022 (33)	Inner Mongolia	Community population	AWGS	——	92	526	7
11	Han et al. 2022 (34)	Urumqi	Inpatients	SARC-F Scale	——	113	695	6
12	Li et al. 2022 (35)	Tianjin	Community population	IWGS (2011)	BIA	115	475	7
13	Zhang et al. 2022 (36)	Dali	Community population	AWGS (2019)	BIA	21	103	8
14	Li et al. 2022 (37)	Zhejiang	Community population	AWGS (2014)	BIA	278	1,420	6
15	Ko et al. 2021 (38)	Taiwan	Health examination population	AWGS (2019)	BIA	138	500	8
16	Chang et al. 2021 (39)	Taiwan	Nursing home population	AWGS (2019)	BIA	88	170	7
17	Pan et al. 2021 (40)	Xining	Inpatients	AWGS (2014)	BIA	30	150	6
18	Zhang et al. 2021 (41)	Jiangsu	Inpatients	AWGS (2019)	BIA	173	445	7
19	Liu et al. 2020 (42)	Western China*	Community population	AWGS	BIA	556	1712	7
20	Yang et al. 2020 (43)	Urumqi	Community population	EWGSOP (2018) EWGSOP (2014) AWGS (2014) IWGS (2011)	BIA	483	22 76 44 78	6
21	Chen et al. 2020 (44)	Nanjing	Community population	EWGSOP	DXA	27	249	7
22	Xu et al. 2020 (45)	Chengdu	Inpatients	AWGS (2014)	DXA	63	142	6
23	Liang et al. 2020 (46)	Beijing	Inpatients	AWGS (2014)	DXA	80	180	8
24	Meng et al. 2020 (47)	Xinxiang	Community population	AWGS (2014)	BIA	131	1,004	7
25	Che et al. 2020 (48)	Urumqi	Community population	Ishii Scale	–	287	740	8
26	Tian et al. 2020 (49)	Urumqi	Community population	AWGS (2014)	BIA	35	395	8
27	Yang et al. 2019 (50)	Suzhou	Nursing home population	AWGS (2014)	BIA	91	316	8
28	Yao et al., 2019 (51)	Xiangtan	Inpatients	AWGS	BIA	47	378	7
29	Liu et al. 2019 (52)	Chongqing	Community population	AWGS (2014)	BIA	14	247	7
30	He et al. 2019 (53)	Beijing	T2DM patients	AWGS (2014)	BIA	75	650	6
31	Hao et al. 2018 (54)	Chengdu	Inpatients	AWGS	BIA	127	407	7
32	Zeng et al. 2018 (55)	Chengdu	Nursing home population	EWGSOP AWGS IWGS	BIA	277	90 95 106	7
33	Zhang et al. 2018 (56)	Shanghai	Community population	AWGS	BIA	164	1,148	8
34	Wang et al. 2018 (57)	Chengdu	Community population	AWGS (2014)	BIA	71	915	7
35	Yang et al. 2018 (58)	Suzhou	Community population	AWGS	DXA	91	316	8
36	Liao et al. 2018 (59)	Chongqing	Nursing home population	AWGS (2014)	BIA	86	225	6
37	Wang et al. 2018 (60)	Nantong	Outpatient	AWGS (2014)	BIA	92	407	8
38	Hai et al. 2017 (61)	Chengdu	Community population	AWGS	BIA	88	836	8
39	Hu et al. 2017 (62)	Sichuan	Community population	AWGS (2014)	DXA	112	607	7
40	Han et al. 2016 (63)	Tianjin	Community population	AWGS (2014)	BIA	99	1,069	6
41	Xia et al. 2016 (64)	Beijing	Community population	AWGS (2014)	BIA	137	683	6
42	Gao et al. 2015 (65)	Sichuan	Community population	AWGS	—	60	612	9
43	Meng et al. 2015 (66)	Taiwan	Community population	EWGSOP	DXA	44	771	7
44	Wu et al. 2014 (67)	Taiwan	Community population	EWGSOP	BIA	39	549	6
45	Yu et al. 2014 (68)	Hong Kong	Community population	EWGSOP	—	361	4,000	8

AWGS, Asian Working Group on Sarcopenia; EWGSOP, European Working Group on Sarcopenia in Older People; IWGS, International Working Group on Sarcopenia; SARC-F Scale, a simple five-item scoring questionnaire for sarcopenia; Ishii Scale, a simple three-item scoring questionnaire for sarcopenia; BIA, bioelectrical impedance method; DXA, dual-energy X-ray absorption method; AHRQ, Agency for Healthcare Research and Quality; T2DM, type 2 diabetes. Yunnan-guizhou Plateau region*, specifically Liupanshui City and Kunming City; Western China*, specifically Yunnan, Guizhou, Sichuan and Xinjiang.

### 3.3 Meta-analysis results

#### 3.3.1 Meta-analysis of the prevalence of sarcopenia among the older adult in China

**Figure 3:**
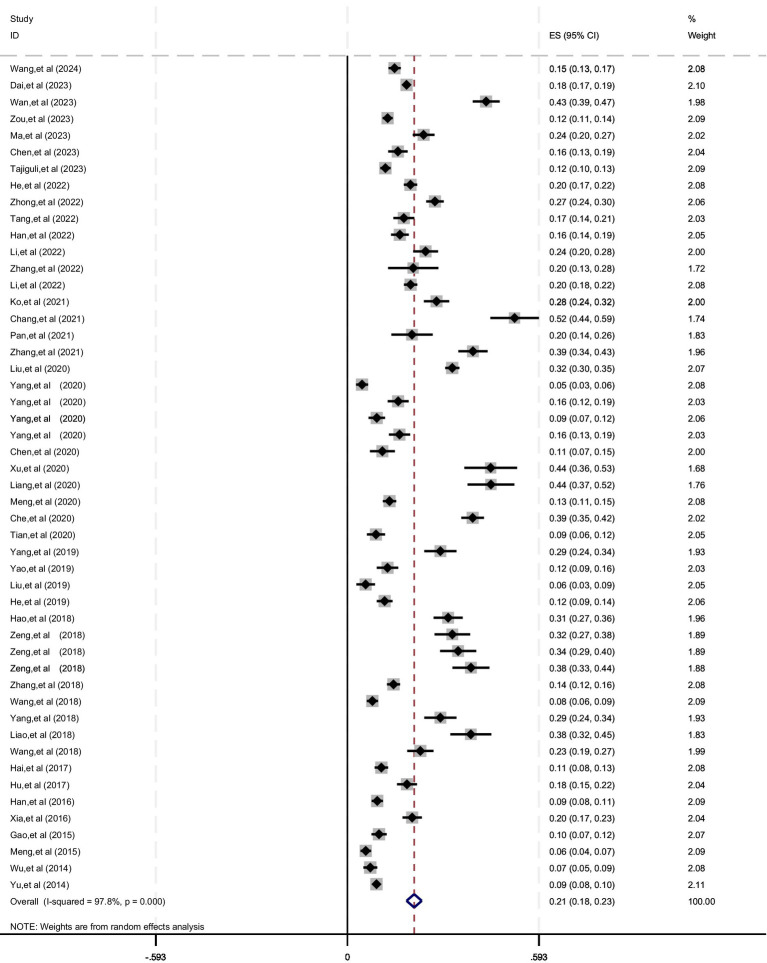
Meta-analysis forest figure of the prevalence of sarcopenia among the older adult in China.

We studied the prevalence of sarcopenia in the older adult in 45 literatures. The result of the meta-analysis showed that *I^2^* = 97.8%, *p* < 0.001, which indicated that the heterogeneity among the studies was high. The sensitivity analysis was carried out by the one-by-one elimination method, and no literature that had a significant impact on the overall results was found, so the random effect model was used for combined analysis. Meta-analysis shows that the prevalence of sarcopenia among the older adult (≥60) in China is 20.7% [95% CI (18.3, 23.0%)], as shown in Figure 3 and Table 3.

**Table 3:** Summary table of meta-analysis results of prevalence of sarcopenia in the older adult in China.

Subgroup	Number of included literature	Results of heterogeneity test		Effect model	The prevalence % (95%Cl)
		*I^2^*	*p*		
Total prevalence rate (24–68)	45	99.2%	<0.001	Random effect model	20.7 (18.3, 23.0)
Sex					
Men (24, 25, 27, 29–33, 35, 37–41, 43–45, 47, 48, 50, 51, 54–64, 66, 67)	34	94.7%	<0.001	Random effect model	19.2 (16.6, 21.8)
Women (24, 25, 27, 29–33, 35, 37–41, 43–45, 47, 48, 50, 51, 54–64, 66, 67)	34	96.5%	<0.001	Random effect model	21.6 (18.7, 24.5)
Age					
60–69 (26, 27, 31–33, 35, 41, 48, 64, 65)	10	96.0%	<0.001	Random effect model	15.7 (10.6, 20.7)
70–79 (26, 27, 31–33, 35, 41, 48, 64, 65)	10	95.4%	<0.001	Random effect model	27.2 (20.2, 34.3)
≥80 (26, 27, 31–33, 35, 41, 48, 64, 65)	10	88.8%	<0.001	Random effect model	45.4 (35.9, 54.9)
Region					
South (24, 29, 31, 32, 36–39, 41, 44, 45, 50–52, 54–62, 65–68)	27	97.7%	<0.001	Random effect model	21.7 (18.5, 24.9)
North (26, 28, 30, 33–35, 40, 43, 46–49, 53, 63, 64)	15	97.6%	<0.001	Random effect model	19.0 (14.9, 23.1)
Publication year					
2014–2018 (54–68)	15	97.3%	<0.001	Random effect model	19.2 (15.8, 22.7)
2019–2024 (24–53)	30	97.6%	<0.001	Random effect model	21.4 (18.4, 24.3)
Diagnostic criteria					
AWGS (2019) (24–32, 36, 38, 39, 41)	13	97.6%	<0.001	Random effect model	24.5 (20.3, 28.7)
AWGS (2014) (37, 40, 43, 45–47, 49, 50, 52, 53, 57, 59, 60, 62–64)	16	96.4%	<0.001	Random effect model	19.3 (15.5, 23.1)

#### 3.3.2 Subgroup analysis of the prevalence of sarcopenia among the older adult in China

Meta-analysis of the prevalence of sarcopenia suggests that there is high heterogeneity among studies. We intend to use subgroup analysis to try to identify the source of clinical heterogeneity. The subgroups in this study included: gender (male; Female), age range (60–69; 70–79; ≥80), region (South/North; With Qinling Mountains and Huaihe River as the boundary, north of Qinling Mountains and Huaihe River is north, and south of Huaihe River is south), publication time (2014–2018; 2019–2024), diagnostic criteria (AWGS2019; AWGS2014). The results of the subgroup analysis are shown in Table 3.

#### 3.3.3 Meta-analysis of the risk factor of sarcopenia among the older adult in China

We included 45 articles related to the risk factors of sarcopenia. After extraction, induction, and data processing by relevant personnel, we found that there were 10 risk factors involved in 2 or more studies. We made a meta-analysis of these risk factors of sarcopenia, and the results showed that aging, low BMI, low leg circumference, smoking, depression, osteoporosis, malnutrition and malnutrition risk were all risk factors of sarcopenia among the older adult in China. The results of the meta-analysis of influencing factors of sarcopenia are shown in Table 4.

**Table 4:** Summary table of meta-analysis results of risk factors of sarcopenia in the older adult in China.

Risk factor	Number of included literature	Results of heterogeneity test		Effect model	Result	
		*I* ^2^	*p*		OR (95%CI)	*p*
Sex (women vs. men) (24–26, 31, 36, 38, 39, 43, 47, 54, 65–68)	14	94.3%	<0.001	Random effect model	1.056 (0.936, 1.191)	0.376
Age (Continuous variable) (24, 25, 29, 32, 38, 43, 45, 49–51, 56, 59, 65, 67, 68)	15	67.5%	<0.001	Random effect model	1.128 (1.117, 1.139)	<0.001*
BMI (Continuous variable) (27, 29, 31, 33, 38, 45, 47, 49–52, 54, 59, 66, 67)	15	82.0%	<0.001	Random effect model	0.706 (0.686, 0.727)	<0.001*
Leg circumference (Continuous variable) (29, 47, 48, 55)	4	89.3%	<0.001	Random effect model	0.743 (0.702, 0.786)	<0.001*
Smoking (Yes vs. NO) (24, 47, 54)	3	66.8%	0.049	Random effect model	2.092 (1.469, 2.977)	<0.001*
Depression (Yes vs. NO) (31, 36, 41, 47)	4	0.0%	0.508	Fixed effect model	2.432 (1.716, 3.447)	<0.001*
Osteoporosis (Yes vs. NO) (34, 41, 49, 59)	4	0.0%	0.980	Fixed effect model	2.778 (1.918, 4.026)	<0.001*
Malnutrition (Yes vs. NO) (32, 55)	2	0.0%	0.587	Fixed effect model	2.656 (1.679, 4.200)	<0.001*
Malnutrition risk (Yes vs. NO) (28, 32, 39)	3	36.9%	0.205	Fixed effect model	2.224 (1.712, 2.891)	<0.001*
Malnutrition and malnutrition risk (Yes vs. NO) (36, 37, 65)	3	89.5%	<0.001	Random effect model	1.641 (1.334, 2.019)	<0.001*

## 4 Discussion

### 4.1 The prevalence of sarcopenia in Chinese older adult

In October 2016, sarcopenia was officially included in the International Classification of Diseases (ICD-10) disease code, marking that sarcopenia has been recognized as a new type of geriatric syndrome and has attracted worldwide attention (69). In 2021, Petermann-Rocha et al. reported on the global prevalence of sarcopenia (4): the prevalence of sarcopenia in people aged 60 and above in the world is about 10–27%. A Korean meta-analysis reported that (5): the prevalence of sarcopenia in the older adult aged 60 years and older in Korea was 13.1%. A similar study in Japan reported that the prevalence of sarcopenia in the older adult (≥60) in Japan was 9.9% (6). Diz et al. (70) reported that the overall prevalence of sarcopenia in older adult people aged 60 years and above in Brazil was 17.0% [95%CI (13.0, 22.0%)]. Our results show that the prevalence of sarcopenia in the older adult in China is 20.7%, which is much higher than in similar Asian countries such as Japan (9.9%) (6) and South Korea (13.01%) (5). The specific reason may be that these Asian countries entered the aging society earlier than China, and have gradually established medical security systems that adapt to their national conditions. In general, there are differences in diet, environment, culture, and ethnically specific genetics among countries and regions, which may be the main reasons for the differences in the prevalence of sarcopenia among countries.

Subgroup analysis showed that there was an association between gender and the incidence of sarcopenia, and the prevalence of sarcopenia was higher in women than in men. Our results are consistent with Dai, Yang et al. (25, 43). The specific reasons may be related to the following reasons: First, the ovarian function of older adult women after menopause decreases, and the content of metabolic hormones (such as estradiol and androgen) in the body will also decrease, which seriously affects the generation of muscle cells and protein synthesis (71). On the other hand, women are more focused on body image management, leading to inadequate nutrient intake, which may also be related to the higher prevalence of sarcopenia in women. However, Chinese scholars Ren, Chen et al. reported the opposite result (17, 19): the prevalence of sarcopenia in older adult Chinese men was higher. They suggest that this difference may be related to the unhealthy lifestyle of men (such as smoking and drinking). As we age, the effects of an unhealthy lifestyle may significantly increase, and these factors can all contribute to sarcopenia (5). In addition, a 12-year cohort study in Japan showed that (72): men were more likely to experience muscle mass loss than women. About this difference, we suggest that future multi-center, large-sample prospective studies can be conducted to continue to explore the relationship between gender and the occurrence of sarcopenia in the older adult, to better implement precise prevention.

Sarcopenia is a well-recognized age-related degenerative condition. With the global rise in aging populations, the prevalence of sarcopenia is expected to increase significantly. This was also confirmed in our study: by age, the prevalence was highest in the older age group ≥80 years (45.4%), followed by 70–79 years (27.2%), and finally 60–69 years (15.7%). Under normal physiological conditions, human skeletal muscle mass reaches its peak at around 25 years of age, and then the number and volume of skeletal muscle fibers begin to decline, and the decline rate gradually accelerates with the increase of age, which is the result of synergistic regulation of multiple aging mechanisms in the human body (1). In addition, with the growth of age, the incidence of various basic diseases in the older adult will also increase, which will also affect the regulation of various hormones and the absorption of nutrients, thereby indirectly affecting the decline of muscle mass (73). Compared with the north (19.0%), the prevalence of sarcopenia was higher in the older adult in the south (21.7%). Our study was consistent with Mao et al. (74), but some studies reported the opposite result (17). China’s vast territory, the climate environment, and the dietary habits of different regions are quite different, which may be the main reason for the difference in prevalence. The climate in the north is mostly cold. Due to the influence of climate, the local people’s diet mainly includes “beef and mutton, dairy products and nuts,” and the incidence of malnutrition and malnutrition risk is relatively low (75). So far, there are few studies on regional differences in the prevalence of sarcopenia in China, which can be further verified with more relevant epidemiological studies in the future.

In addition to the differences in demographic characteristics, the different diagnostic criteria of sarcopenia also directly affect the detection rate of sarcopenia. Based on the fact that China is in Asia, and to ensure the comparability of this study in Asian populations, we selected AWGS for subgroup analysis in terms of diagnostic criteria. Compared with AWGS (2014), the latest AWGS (2019) has significantly improved the evaluation criteria of walking speed and male grip strength (among which, walking speed has been increased from 0.8 m/s to 1.0 m/s, and male grip strength has been increased from 26 kg to 28 kg). As a result, the AWGS (2019) will have a higher rate of sarcopenia detection, and our study also confirms this result. Domestic studies on the evaluation of sarcopenia in the older adult based on AWGS (2014) criteria also generally reported a low incidence [Xinxiang 13.1% (47), Chengdu 10.6% (61), Tianjin 9.3% (63)]. Finally, in the last 5 years, the prevalence of sarcopenia in the older adult in China has been on the rise. It increased from 19.2% in 2014–2018 to 21.4% in 2019–2024. We infer that this difference may be due to past and present differences in health and medical resources. At the same time, the widening of diagnostic criteria may also be one of the reasons for the significant increase in the prevalence of sarcopenia reported in China in recent years.

### 4.2 The risk factor of sarcopenia in Chinese older adult

The occurrence of sarcopenia in the older adult is related to many factors. Our study shows that aging, low BMI, low calf circumference, smoking, depression, osteoporosis, malnutrition and malnutrition risk are risk factors for sarcopenia in the older adult in China.

There is no doubt that aging is one of the risk factors for sarcopenia. Pang et al. reported the epidemiology of sarcopenia in the Singapore community population (76): the prevalence of sarcopenia was 13.6% in the community as a whole (21–90 years), but 32% in people over 60 years of age. A study in Thailand also found that (77): older adult people (≥80 years old) had the highest prevalence of sarcopenia (68%). A 12-year prospective population study in Sweden showed that (78): even subjects without sarcopenia had a 5.1% probability of developing suspected sarcopenia over 10 years. This is consistent with our findings that the older people get, the higher their risk of sarcopenia.

Secondly, bad lifestyle habits can also affect the prevalence of sarcopenia, of which smoking is considered to be a risk factor for sarcopenia. Previous studies have reported that the risk of sarcopenia in older adult smokers in Asia is 2.69 times that of non-smokers (79). The risk of sarcopenia among older smokers in Europe is 2.36 times that of non-smokers (80). We speculate that the reason may be related to the following reasons: on the one hand, smoking directly damages the health of skeletal muscle. Smoking can damage muscle metabolism, increase inflammation and oxidative stress, increase the overexpression of genes related to muscle atrophy, and activate various intracellular signaling pathways, thus causing skeletal muscle injury (81). On the other hand, smoking will increase the risk of cancer, respiratory diseases, and cardiovascular and cerebrovascular diseases (82), thus increasing energy consumption and reducing the activity capacity of the older adult, indirectly leading to the occurrence of sarcopenia. Therefore, not smoking or quitting early may be important for the prevention and treatment of sarcopenia.

There is also a link between depression, osteoporosis, and sarcopenia. Turkish scholar Olgun-Yazar et al. pointed out that (83): depression and sarcopenia are closely related. Symptoms associated with depression, such as weakness, loss of appetite, and decreased activity, may contribute to the onset and progression of sarcopenia (84). Meanwhile, some inflammatory cytokines secreted by the musculoskeletal system are closely related to the occurrence of depression, such as IL-6, TNF-a, and 5-HT (85). In the early prevention and treatment of sarcopenia, it is necessary to pay attention to and care for the depressed older adult.

Osteoporosis is a systemic bone metabolic disease characterized by low bone mass and mass, which can increase susceptibility to sarcopenia (86). Sarcopenia and osteoporosis are both associated with aging, low quality of life, and low condition of health, and they often occur together. At present, some scholars have shown that there is a close relationship between muscle and bone, namely, osteosarcopenia, and sarcopenia-osteoporosis (87, 88). Maurel et al. (89) investigated the relationship between sarcopenia and osteoporosis and showed that osteoporosis increases the risk of developing sarcopenia. Our study also found that osteoporosis is a risk factor for sarcopenia. Specific reasons may be related to the connection between muscle and bone. For example, Bone cells secrete osteocalcin, insulin-like, etc., affecting muscle quality and function, and the decrease of bone cells will lead to the decline of muscle mass and function (90). Therefore, we should pay attention to the role of bone health in the occurrence of sarcopenia, and reduce the incidence of sarcopenia while maintaining bone health in the older adult.

Finally, there is also a close relationship between the nutritional status of the body and sarcopenia (91). BMI and calf circumference can be used to evaluate the nutritional status of the older adult. Among them, calf circumference is currently recognized as an alternative marker of muscle mass and is used as one of the diagnostic criteria for sarcopenia (92, 93). To a certain extent, BMI can also reflect the nutritional status of the body. A higher BMI is a protective factor for sarcopenia and is positively correlated with muscle mass (94). It is worth noting that there is a special condition here, that is, sarcopenic obesity. This is a condition that occurs based on excessive obesity, with decreased muscle tissue and increased fat infiltration between muscle fibers and muscle cells (10, 12). Medical and health workers should pay attention to health education: the older adult can properly maintain a high BMI level under the premise of not obesity. In addition, physiological evidence shows that when the body is in poor nutritional condition, amino acids, and muscles are broken down and oxidized to produce energy to maintain normal life functions. If sustained for a longer period, it will result in a negative nitrogen balance, as well as a gradual loss of muscle mass and function (95). In addition, poor nutritional status can also lead to micronutrient deficiencies (such as vitamin D and vitamin B12), which further leads to muscle loss and decreased function (96, 97). In conclusion, timely screening and assessment of the nutritional status of the older adult may be an effective strategy for early detection, diagnosis, and management of patients with sarcopenia. Proven and easy-to-use nutrition screening tools are necessary, such as a simple nutritional assessment profile (Mini Nutritional Assessment Short Form, MNA – SF) can be used to determine the nutritional status of the older adult and the poor nutrition risk (98).

So far, this study is currently the most geographically diverse and has the highest number of subjects in the epidemiological study of sarcopenia in older adult people in China, and involves risk factors for sarcopenia. We reported the latest data and risk factors for sarcopenia in older adult people aged 60 and above in China. We conducted differences in gender, age, region (southern/northern), diagnostic criteria, and other factors through subgroup analysis. These works provide more information and evidence-based evidence for the epidemiology of sarcopenia and are the first step in developing preventive measures or health services for the older adult. In addition, our study also found that aging, low BMI, low calf circumference, smoking, depression, osteoporosis, malnutrition and malnutrition risk are risk factors for the development of sarcopenia in the older adult in China. For the older adult population with high-risk factors, timely prevention and screening is very necessary.

There are some limitations to the study. First, we collected representative data for each region but were limited by the characteristics of individual studies and differences between different cities, which may affect comparisons between included studies. Secondly, some studies reported prevalence rates for different age groups and different risk factors, and relevant data could not be included in subgroups, resulting in fewer studies included in some subgroups (such as smoking, malnutrition, and malnutrition, etc. risk factors), which may also affect comparisons between included studies. Finally, this study is based primarily on evidence from observational studies that cannot provide information about causation for the observed associations. Currently, there is a lack of high-quality prospective cohort studies on sarcopenia, especially in exploring the risk factors for sarcopenia. In addition to focusing on clinical patients susceptible to sarcopenia, cohort studies with accurate measurements of muscle quantity and function in the general healthy population are needed to provide evidence for developing primary prevention strategies.

## 5 Conclusion

The results of this study show that the overall prevalence of sarcopenia in the older adult aged 60 and above in China is relatively high (20.7%), and it shows a gradual increasing trend in the past 5 years. This phenomenon should arouse the attention and concern of public health departments. Finally, we suggest that the public health sector should carry out early screening, intervention, and management of sarcopenia promptly, especially for the older adult population with high-risk factors (such as osteoporosis and depression).

## Data availability statement

The original contributions presented in the study are included in the article/Supplementary material, further inquiries can be directed to the corresponding author.

## Author contributions

SM: Writing – original draft, Writing – review & editing, Data curation, Formal analysis, Investigation, Project administration, Software. XH: Writing – original draft, Data curation, Investigation, Software, Writing – review & editing. XF: Conceptualization, Writing – review & editing, Data curation, Formal analysis, Software. XZ: Writing – review & editing, Data curation, Software. MT: Writing – review & editing, Data curation, Project administration, Software, Supervision, Validation. WL: Writing – original draft, Writing – review & editing, Data curation, Methodology, Software. WZ: Writing – original draft, Conceptualization, Data curation, Formal analysis, Software. XS: Writing – original draft, Writing – review & editing, Conceptualization, Data curation, Formal analysis, Investigation, Methodology, Software, Supervision. KL: Conceptualization, Data curation, Formal analysis, Funding acquisition, Investigation, Methodology, Project administration, Resources, Software, Supervision, Validation, Visualization, Writing – original draft, Writing – review & editing.

## Supplementary material

SUPPLEMENTARY FILE 1 The detailed information of literature retrieval strategy.

SUPPLEMENTARY FILE 2 The detailed results of literature evaluation.

SUPPLEMENTARY FILE 3 The general information of included literature.

SUPPLEMENTARY FILE 4 The corresponding PRISMA checklist.
