# Supplementary material for: The prevalence of sarcopenia and risk factors in the older adult in China: a systematic review and meta-analysis
Source: Front Public Health. 2024 Aug 5;12:1415398. doi: 10.3389/fpubh.2024.1415398 (PMC11331796; doi:10.3389/fpubh.2024.1415398)
Supplement: SUPPLEMENTARY FILE 1 — The detailed information of literature retrieval strategy. [file Table_1.DOCX]

The following is the database search strategy of this study.

**Search strategy**

P（population）：Chinese elderly ≥60 years old

I（intervention）：no interventions

C（comparison）：no comparisons

O（outcome）： Prevalence and risk factors of sarcopenia in Chinese elderly

S（study）：Cross-sectional study or Retrospective study

**Search formula**

1. The search on **PubMed, Web of Science, Embase, and Cochrane Library** was performed with the following search formula:

("sarcopenia"[MeSH Terms] OR "sarcopenia"[All Fields] OR "sarcopenia s"[All Fields]) AND ("epidemiology"[MeSH Subheading] OR "epidemiology"[All Fields] OR "prevalence"[All Fields] OR "prevalence"[MeSH Terms] OR "prevalance"[All Fields] OR "prevalences"[All Fields] OR "prevalence s"[All Fields] OR "prevalent"[All Fields] OR "prevalently"[All Fields] OR "prevalents"[All Fields] OR ("epidemiology"[MeSH Subheading] OR "epidemiology"[All Fields] OR "incidence"[All Fields] OR "incidence"[MeSH Terms] OR "incidences"[All Fields] OR "incident"[All Fields] OR "incidents"[All Fields]) OR ("epidemiologies"[All Fields] OR "epidemiology"[MeSH Subheading] OR "epidemiology"[All Fields] OR "epidemiology"[MeSH Terms] OR "epidemiology s"[All Fields])) AND (("risk factors"[MeSH Terms] OR ("risk"[All Fields] AND "factors"[All Fields]) OR "risk factors"[All Fields]) AND (("correlate"[All Fields] OR "correlated"[All Fields] OR "correlates"[All Fields] OR "correlating"[All Fields] OR "correlation"[All Fields] OR "correlation s"[All Fields] OR "correlations"[All Fields] OR "correlative"[All Fields] OR "correlatives"[All Fields]) AND ("factor"[All Fields] OR "factor s"[All Fields] OR "factors"[All Fields])) AND (("affect"[MeSH Terms] OR "affect"[All Fields] OR "affects"[All Fields] OR "affected"[All Fields] OR "affecteds"[All Fields] OR "affecting"[All Fields]) AND ("factor"[All Fields] OR "factor s"[All Fields] OR "factors"[All Fields]))) AND ("china"[MeSH Terms] OR "china"[All Fields] OR "china s"[All Fields] OR "chinas"[All Fields] OR ("chineses"[All Fields] OR "east asian people"[MeSH Terms] OR ("east"[All Fields] AND "asian"[All Fields] AND "people"[All Fields]) OR "east asian people"[All Fields] OR "chinese"[All Fields])).

2. The search on **CNKI, Wan Fang Data, and VIP** was performed with the following search formula:

（（肌肉减少症[主题词]）或（肌少症[主题词]）或（少肌症[主题词]）或（骨骼肌减少症[主题词]））和（（患病率[主题词]）或（发病率[主题词]）或（流行病学[主题词]））和（影响因素[主题词]）或（相关因素[主题词]）或（危险因素[主题词]））
