# Supplementary material for: The prevalence of sarcopenia and risk factors in the older adult in China: a systematic review and meta-analysis
Source: Front Public Health. 2024 Aug 5;12:1415398. doi: 10.3389/fpubh.2024.1415398 (PMC11331796; doi:10.3389/fpubh.2024.1415398)
Supplement: SUPPLEMENTARY FILE 2 — The detailed results of literature evaluation. [file Table_2.DOCX]

Table 1. Results of quality evaluation included in the literature

| **Study** | **Q1** | **Q2** | **Q3** | **Q4** | **Q5** | **Q6** | **Q7** | **Q8** | **Q9** | **Q10** | **Q11** | **Total score** | **Rate** |
| --- | --- | --- | --- | --- | --- | --- | --- | --- | --- | --- | --- | --- | --- |
| Wang.et al,2024 | 1 | 1 | 1 | 1 | 1 | 0 | 1 | 1 | 0 | 1 | 0 | 8 | High |
| Dai.et al,2023 | 1 | 1 | 0 | 0 | 1 | 1 | 1 | 1 | 0 | 0 | 0 | 6 | Middle |
| Wan.et al,2023 | 1 | 1 | 1 | 1 | 0 | 1 | 0 | 1 | 0 | 0 | 0 | 6 | Middle |
| Zou.et al,2023 | 1 | 1 | 1 | 1 | 1 | 0 | 0 | 1 | 0 | 0 | 0 | 6 | Middle |
| Ma.et al,2023 | 1 | 1 | 1 | 1 | 0 | 1 | 0 | 1 | 0 | 0 | 0 | 6 | Middle |
| Chen.et al,2023 | 1 | 1 | 1 | 1 | 1 | 1 | 0 | 1 | 0 | 0 | 0 | 7 | Middle |
| Musha.et al,2023 | 1 | 1 | 1 | 0 | 1 | 1 | 0 | 1 | 0 | 1 | 0 | 7 | Middle |
| He.et al,2022 | 1 | 1 | 1 | 1 | 1 | 1 | 1 | 1 | 0 | 1 | 0 | 9 | High |
| Zhong.et al,2022 | 1 | 1 | 1 | 1 | 1 | 1 | 0 | 1 | 0 | 0 | 0 | 7 | Middle |
| Tang.et al,2022 | 1 | 1 | 1 | 1 | 1 | 1 | 0 | 0 | 0 | 1 | 0 | 7 | Middle |
| Han.et al,2022 | 1 | 1 | 1 | 1 | 0 | 1 | 0 | 0 | 0 | 1 | 0 | 6 | Middle |
| Li.et al,2022 | 1 | 1 | 1 | 1 | 1 | 1 | 0 | 1 | 0 | 0 | 0 | 7 | Middle |
| Zhang.et al,2022 | 1 | 1 | 1 | 1 | 1 | 1 | 0 | 1 | 0 | 1 | 0 | 8 | High |
| Li.et al,2022 | 1 | 1 | 1 | 1 | 1 | 1 | 0 | 0 | 0 | 0 | 0 | 6 | Middle |
| Ko.et al,2021 | 1 | 1 | 1 | 1 | 1 | 0 | 1 | 1 | 0 | 1 | 0 | 8 | High |
| Chang.et al,2021 | 1 | 1 | 1 | 1 | 1 | 0 | 1 | 1 | 0 | 0 | 0 | 7 | Middle |
| Pan.et al,2021 | 1 | 1 | 1 | 1 | 0 | 1 | 0 | 1 | 0 | 0 | 0 | 6 | Middle |
| Zhang.et al,2021 | 1 | 1 | 1 | 1 | 1 | 1 | 0 | 1 | 0 | 0 | 0 | 7 | Middle |
| Liu.et al,2020 | 1 | 0 | 1 | 0 | 1 | 1 | 1 | 1 | 0 | 1 | 0 | 7 | Middle |
| Yang.et al,2020 | 1 | 1 | 1 | 1 | 1 | 1 | 0 | 0 | 0 | 0 | 0 | 6 | Middle |
| Chen.et al,2020 | 1 | 1 | 1 | 1 | 1 | 1 | 0 | 1 | 0 | 0 | 0 | 7 | Middle |
| Xu.et al,2020 | 1 | 1 | 1 | 0 | 1 | 1 | 0 | 1 | 0 | 0 | 0 | 6 | Middle |
| Liang.et al,2020 | 1 | 1 | 1 | 1 | 1 | 1 | 0 | 1 | 0 | 1 | 0 | 8 | High |
| Meng.et al,2020 | 1 | 1 | 1 | 0 | 1 | 1 | 0 | 1 | 0 | 1 | 0 | 7 | Middle |
| Che.et al,2020 | 1 | 1 | 1 | 1 | 1 | 1 | 0 | 1 | 0 | 1 | 0 | 8 | High |
| Tian.et al,2020 | 1 | 1 | 1 | 1 | 1 | 1 | 0 | 1 | 0 | 1 | 0 | 8 | High |
| Yang.et al,2019 | 1 | 1 | 1 | 1 | 1 | 1 | 0 | 1 | 0 | 1 | 0 | 8 | High |
| Yao.et al,2019 | 1 | 1 | 1 | 1 | 1 | 1 | 0 | 1 | 0 | 0 | 0 | 7 | Middle |
| Liu.et al,2019 | 1 | 1 | 1 | 1 | 1 | 1 | 0 | 1 | 0 | 0 | 0 | 7 | Middle |
| He.et al,2019 | 1 | 1 | 1 | 0 | 1 | 1 | 0 | 1 | 0 | 0 | 0 | 6 | Middle |
| Hao.et al,2018 | 1 | 1 | 1 | 0 | 1 | 0 | 1 | 1 | 0 | 1 | 0 | 7 | Middle |
| Zeng.et al,2018 | 1 | 1 | 1 | 1 | 1 | 1 | 0 | 1 | 0 | 0 | 0 | 7 | Middle |
| Zhang.et al,2018 | 1 | 1 | 1 | 1 | 1 | 1 | 0 | 1 | 0 | 1 | 0 | 8 | High |
| Wang.et al,2018 | 1 | 1 | 1 | 1 | 1 | 1 | 0 | 0 | 0 | 1 | 0 | 7 | Middle |
| Yang.et al,2018 | 1 | 1 | 1 | 1 | 1 | 1 | 0 | 1 | 0 | 1 | 0 | 8 | High |
| Liao.et al,2018 | 1 | 1 | 1 | 1 | 1 | 0 | 0 | 1 | 0 | 0 | 0 | 6 | Middle |
| Wang.et al,2018 | 1 | 1 | 1 | 1 | 1 | 1 | 0 | 1 | 0 | 1 | 0 | 8 | High |
| Hai.et al,2017 | 1 | 1 | 1 | 1 | 1 | 1 | 1 | 1 | 0 | 0 | 0 | 8 | High |
| Hu.et al,2017 | 1 | 1 | 1 | 0 | 0 | 1 | 1 | 1 | 0 | 1 | 0 | 7 | Middle |
| Han.et al,2016 | 1 | 1 | 1 | 0 | 1 | 1 | 0 | 1 | 0 | 0 | 0 | 6 | Middle |
| Xia.et al,2016 | 1 | 1 | 0 | 0 | 1 | 1 | 0 | 1 | 0 | 1 | 0 | 6 | Middle |
| Gao.et al,2015 | 1 | 1 | 1 | 0 | 1 | 1 | 1 | 1 | 1 | 1 | 0 | 9 | High |
| Meng.et al,2015 | 1 | 0 | 0 | 1 | 1 | 1 | 1 | 1 | 0 | 1 | 0 | 7 | Middle |
| Wu.et al,2014 | 1 | 1 | 0 | 0 | 1 | 0 | 1 | 1 | 0 | 1 | 0 | 6 | Middle |
| Yu.et al,2014 | 1 | 0 | 1 | 1 | 1 | 1 | 0 | 1 | 1 | 0 | 1 | 8 | High |
